# Non-Coding RNAs as Regulators of Myogenesis and Postexercise Muscle Regeneration

**DOI:** 10.3390/ijms222111568

**Published:** 2021-10-26

**Authors:** Karolina Archacka, Maria A. Ciemerych, Anita Florkowska, Karolina Romanczuk

**Affiliations:** Department of Cytology, Institute of Developmental Biology and Biomedical Sciences, Faculty of Biology, University of Warsaw, Miecznikowa 1, 02-096 Warsaw, Poland; kczaja@biol.uw.edu.pl (K.A.);a.florkowska@biol.uw.edu.pl (A.F.); k.romanczuk@biol.uw.edu.pl (K.R.)

**Keywords:** mammals, human, mouse, miRNA, lncRNA, myogenesis, regeneration, training, skeletal muscle

## Abstract

miRNAs and lncRNAs do not encode proteins, but they play an important role in the regulation of gene expression. They differ in length, biogenesis, and mode of action. In this work, we focus on the selected miRNAs and lncRNAs involved in the regulation of myogenesis and muscle regeneration. We present selected miRNAs and lncRNAs that have been shown to control myogenic differentiation and show that manipulation of their levels could be used to improve myogenic differentiation of various types of stem and progenitor cells. Finally, we discuss how physical activity affects miRNA and lncRNA expression and how it affects muscle well-being.

## 1. So Many of Them....

The discovery of noncoding RNAs (ncRNAs) has presented new perspectives for understanding the mechanisms regulating gene expression and cell functions. The long list of ncRNAs includes ribosomal RNA (rRNA), transfer RNA (tRNA), small nuclear RNA (snRNA), small nucleolar RNA (snoRNA), circular RNA (circRNA), microRNA (miRNA), and long noncoding RNA (lncRNA) (reviewed in [1]). The first ncRNAs, i.e., tRNAs and rRNAs, were described in the 1950s [2]. Next, snoRNAs involved in rRNA processing, snRNAs engaged in splicing, and other types of RNA were described [3,4]. A growing group of already known ncRNAs was soon enlarged by small interfering RNAs (siRNAs), PIWI-interacting RNAs (piRNAs), miRNAs, and finally lncRNAs (for the detailed review see [1,2]). Here, we focus only on two groups of ncRNAs: miRNAs and lncRNAs, and their generation and function in skeletal muscle. Due to the enormous and fast-growing amount of data available, we highlight only selected molecules involved in myogenic differentiation and skeletal muscle function, with special emphasis on their role in the muscle response to physical activity.

## 2. miRNA and lncRNA Biogenesis and Mode of Action

### 2.1. miRNA

miRNAs are short molecules composed of approximately 18 to 30 nucleotides. Each of them contains a unique seed sequence that allows one to recognize target mRNAs. Other sequences, however, could be involved in miRNA/mRNA interactions. Next, miRNAs contain additional domains at 5′ and 3′ ends responsible for their interaction with other factors in the miRNA processing machinery. These features allow one to identify and foresee multiple molecules belonging to the miRNA group. The first miRNA to be described was lin-4. Since its discovery in *Caenorhabditis elegans* [5], thousands of these molecules have been identified and their function verified, or at least predicted. In 2018, public MirBase listed 38,589 entries (https://www.mirbase.org/ accessed on 26 October 2021) [6]. Importantly, many tools (bioinformatics) allowing for miRNA prediction and analysis are currently available [7].

miRNA biogenesis is a multistep and begins with transcription catalyzed by polymerase II [8]. miRNA-coding genes are often transcribed as polycistronic pri-miRNA. In the cell nucleus, they are cleaved by DROSHA acting together with DGCR8. The necessity of these enzymatic machines has been proven by generating knock-out mice or cells lacking *Dcgr8* [9,10,11]. *Dcgr8*-null mice were embryonic lethal, and in vitro cultured cells were unable to propagate. DROSHA/DCGR8 mediated cleavage results in the generation of pre-miRNA characterized by hairpin structure [12,13]. Some miRNAs, however, are generated without DROSHA action. They are encoded within so-called mirtrons, which arise from the spliced introns of the host gene mRNA [14]. In other words, such pre-miRNA is generated as a result of splicing. First mirtrons were described in *Drosophila melanogaster* and *Caenorhabditis elegans* [15,16] and then identified in mammals [17]. Despite the method of pre-miRNA generation, they are transported to the cytoplasm with the help of exportin XPO5, where the hairpin is processed by DICER endonuclease [9,18,19]. As a result, double-stranded miRNA is created. Further processing leads to the degradation of one strand and the creation of a functional mature single-stranded miRNA. Many lines of evidence have documented that both strands could be functional. Targeting specific mRNAs by miRNA is based on the sequence complementarity of the miRNA seed (nucleotides 2–7) and 3′ untranslated region (3′UTR) of target mRNAs (e.g., [20,21,22,23]). AGO protein (argonaute) interacts with miRNAs, protecting them from degradation and together with GW182 protein forms ribonucleoprotein complexes—RISC (RNA-induced silencing complexes), which are responsible for gene expression silencing [24,25]. Mature miRNA complexed with RISC interacts with the 3′ UTR of target mRNAs, which leads to mRNA degradation and translational repression. It was also shown to be able to interact with other regions, including the 5′ UTR coding sequence, and gene promoters. Under certain conditions, miRNAs can also activate translation or regulate transcription (e.g., [26,27]).

### 2.2. lncRNA

lncRNAs are a heterogeneous group of regulatory ncRNAs. They are characterized by a minimum size of 200 nucleotides and, unfortunately, very few common features. In 2018, LNCipedia listed as many as 127,802 transcripts and 2482 published articles concerning lncRNAs. In 2021, lncBook included as many as 268,848 molecules. Very few of them have been well characterized so far. lncRNAs are generated via pathways similar to mRNAs and impact gene expression by various modes of action. They can act both in the nucleus and in the cytoplasm. In nucleus, lncRNA function can be executed via several modes (for a detailed review see [1,2]). Their own transcription can interfere with the expression of other genes overlapping target gene promoters (e.g., [28]). They can also affect gene expression by binding enhancer sites or transcription factors (e.g., [29,30]) or recruiting histone-modifying complexes (e.g., [31]). lncRNAs were also shown to be involved in the regulation of nucleus architecture, e.g., organization of chromosome domains, loops, and paraspeckles (e.g., [32,33,34]). Those lncRNAs that act in the cytoplasm influence gene expression by modulating the stability or accessibility of mRNAs [2]. Finally, lncRNAs can bind and sequester miRNAs, preventing them from regulating miRNA- dependent gene silencing (e.g., [35,36,37]). Such molecular “sponges” regulate the distribution and availability of miRNA molecules and thereby impose an additional level of post-transcriptional regulation.

## 3. Examples of miRNAs and lncRNAs Involved in Myogenesis Regulation

As for many other biological processes, the number of miRNAs and lncRNAs involved in the regulation of myogenesis is constantly growing. New data extended the knowledge regarding the network of reciprocal interactions between myogenesis regulators and clearly modified it when contradictory to previous findings. Due to an enormous and fast-growing amount of available data, we describe only selected information and examples of ncRNAs involved in myogenesis. For further information, see [38,39,40,41].

### 3.1. miRNA

Myogenesis is a multistep process controlled by numerous molecules, with key roles played by PAX3 and PAX7 transcription factors and muscle regulatory factors (MRFs), i.e., MYOD (myogenic differentiation 1), MYF5 (myogenic factor 5), MRF4 (myogenic regulatory factor 4), and MYOG (myogenin). Briefly, the proliferation of myogenic cells is connected with the high level of PAX3 and PAX7, while drop in their expression accompanies the differentiation of these cells, together with the increase in MRF expression (for details see [42]).

The network of molecules regulating myogenesis is further filled with numerous ncRNAs. The importance of miRNA activity was demonstrated by O’Rourke et al., who revealed that lack of DICER leads to impairment of embryonic myogenesis resulting in mice death after birth [43]. Then, a few miRNAs, such as miR-1, miR-133a, miR-133b, and miR-206, whose expression is controlled by muscle-related transcription factors, such as MRFs, MEF2, or serum response factor (SRF) [44,45,46,47,48,49], have been described as muscle specific miRNAs, so-called MyomiRs (Table 1) [44,45]. miR-1 and miR-133 are synthesized both in cardiac and skeletal muscles, while miR-206 is exclusively expressed in skeletal muscle [50,51]. miR-1 and miR-206 have been shown to promote myogenesis by inhibiting PAX3 and PAX7 expression [50,51,52,53] and reducing the levels of MRF inhibitors, such as ID1–3 [54]. They block the expression of HDAC4 (histone deacetylase 4), an inhibitor of MEF2, which leads to activation of this transcription factor and initiation of myogenic differentiation [55]. In contrast, miR-133 limits myogenic cell differentiation by inhibiting SRF. Moreover, it promotes myoblast proliferation by inhibiting the expression of cyclin D1, which activates CDK4/6 and controls G1 phase of the cell cycle [46,47,56]. Diverse miR-206 action encompasses targeting POLA1, a subunit of DNAα polymerase, which results in cessation of myogenic cell proliferation, leading to their differentiation [54]. Next, miR-206 was also described to suppress expression of utrophin, which is replaced by dystrophin during skeletal muscle differentiation [49]. These observations were not supported by Liu and co-workers, who did not notice any differences in proliferation nor utrophin expression between wild-type and miR-206 knockout mice [57]. However, the authors confirmed that miR-206 promotes myogenic cell differentiation by suppressing factors preventing its progression, i.e., PAX7 as well as NOTCH3 and IGFBP5 [57]. This stays in line with the report by Przanowska et al., 2020, who revealed that triple knockout mice devoid of miR206/miR1a1/miR1a2 are characterized by a higher number of PAX7 positive cells [58].

Apart from MyomiRs, also other non-muscle-specific miRNAs are involved in the formation and functioning of skeletal muscles. Among them are miR-27b, miR-181a, miR-214, and miR-486 (Table 1). miR-181a expression increases during myogenic differentiation and promotes it by targeting HOXA11 (homeobox protein A11), an inhibitor of *MyoD* expression [61]. Next, miR-214 influences myogenesis by decreasing the expression of *N-ras*, which inhibits p21CIP1 expression. As a result of miR-214 action, p21CIP1 becomes active in cells, ceasing their proliferation and thus promoting differentiation [62]. Similarly to miR-1 and miR-206, miR-486 inhibits *Pax7* [51] while miR-27b inhibits *Pax3*. Expression of miR-27b is observed in cells differentiating in the myotome, as well as in activated satellite cells within adult skeletal muscle. The complex interactions of miRNAs (including MyomiRs) and muscle-specific molecules (e.g., MRFs) create a subtle balance vital for proper muscle development and growth.

### 3.2. lncRNAs

Fate of myogenic cells is also regulated by numerous lncRNAs (for example, see Table 2). The first lncRNA described as myogenesis regulator was H19, which—unlike other lncRNAs—is evolutionary highly conserved. H19, whose expression is induced by MYOD, is expressed in many embryonic tissues, but after birth it is specifically expressed in skeletal muscles only and its level increases during muscle regeneration [65]. Silencing of *H19* by siRNA treatment resulted in decreased expression of numerous genes necessary for myogenic differentiation, including *Myog* and *Myhc*, which hampered this process [35]. It has been also revealed that exon 1 of *H19* gene encodes two miRNAs, i.e., miR-675-3p and miR-675-5p. The latter one inhibits the expression of *Cdc6* encoding factor involved in the initiation of DNA replication, while miR-675-3p downregulates SMAD1 and SMAD5 and thus influences BMP signaling pathway. As a result of such action, myogenic differentiation is promoted [35]. Different mode of H19 action depends on their molecular sponge activity towards let-7, the miRNA, which is an upstream regulator of *Igf2* (insulin-like growth factor 2) expression. By limiting the action of let-7, H19 downregulates *Igf2* and thus prevents premature myogenic differentiation [66]. Borensztein et al. revealed reciprocal interactions between IGF2 and H19, showing that *Igf2* gene encodes miR-483, which inhibits SRF expression and consequently downregulates MYOD. Since MYOD enhances H19 expression, its downregulation mediated by IGF2 and miR-483 limits H19 expression. Such negative-feedback loop between MYOD and IGF2 exists during embryonic myogenesis. It manifests in KO mice characterized by a defect in the terminal differentiation of muscle progenitor cells leading to severe diaphragm atrophy and neonatal lethality [65].

In the neighborhood of H19 and IGF2 genes, there is a chromatin region called *nctc* interacting with other muscle-specific lncRNA called Charme (Chromatin architect of muscle expression) [67]. Expression of Charme is restricted to skeletal and cardiac muscles, while its mode of action relies on interaction with several chromatin regions, including *nctc* locus. The latter encodes such muscle-specific genes as *Tnni2* or *Tnnt3,* whose expression is affected by Charme depletion, similarly to, e.g., expression of *Mck* and *Myhc,* which are downregulated after Charme knockdown. As a result of it, mice lacking Charme are characterized by the decrease in muscle fiber size as well as morphological and functional alterations of heart. Thus, Charme is a nuclear lncRNA induced upon myogenic differentiation and promoting later stages of this process.

Among other myogenesis involved in lncRNAs is linc-MD1 (long intergenic noncoding RNA). It is present in the cytoplasm of differentiating myogenic cells and newly regenerated myofibers. It is, however, absent from mature myofibers [36]. linc-MD1 is a competitive endogenous RNA for miR-133 and miR-135. These miRNAs are responsible for blocking the expression of transcription factors MAML1 (mastermind-like protein 1) and MEF2C (myocyte enhancer factor 2C), which are crucial for myogenic differentiation. Interestingly, linc-MD1 sequence encodes miR-133b. At early phases of myogenic differentiation, linc-MD1 binds to HuR protein, which favors its accumulation as well as represses DROSHA mediated cleavage. When myoblast differentiation proceeds miR-133 it becomes upregulated, HuR transcription is blocked, and linc-MD1 is downregulated [68]. Therefore, interaction between HuR, linc-MD1, miR-133, miR-135, MAML1, and MEF2 impacts the progression of myogenic differentiation from early to more advanced stages.

Another lncRNA, i.e., lnc-mg, supports myogenic differentiation. It is localized in the cytoplasm, where it acts as a molecular sponge for miR-351-5p as well as for miR-125b. Such lnc-mg activity leads to miR-351-5p inhibition as well as the increase of this miRNA target, i.e., LACTβ (lactamase β). This prevents proliferation and supports differentiation of in vitro cultured myoblasts. In vivo, it results in muscle hypertrophy and increased number of slow-twitch fibers in mouse gastrocnemius muscles [79]. Moreover, by competing with miR-125b, lnc-mg increases IGF-2 expression and supports myogenic differentiation [72]. Myoparr is another lncRNA supporting myogenic differentiation [75]. This lncRNA is encoded within the MYOG gene (both human and mouse). Its molecular action relies on interaction with DDX17, the coactivator of MYOD, as well as the regulation of association of DDX17 with P300/CBP-associated factor (PCAF) histone acetyltransferase. Myoparr stimulates *Myog* expression, as well as expression of miRNAs regulating cell cycle withdrawal. Thus, it limits the proliferation of myoblasts and promotes their differentiation. *Myog* expression is also regulated by other lncRNA, i.e., DRR eRNA, which is encoded within the enhancer region of *MyoD.* This enhancer region also encodes the second lncRNA—CE eRNA. CE eRNA acts in cis, influencing the expression of its host gene—*MyoD* [69,70]. In contrast, DRR eRNA, which is located on mouse chromosome 7, acts in trans and impacts the expression of *Myog* located in mouse chromosome 1 [69,80]. DRR eRNA binds to *Myog* transcripts and is required for the association and maintenance of cohesin complexes in the gene locus [71]. Among the lncRNAs engaged in myogenesis regulation is also linc-YY1, which appears as a result of reverse transcription from *Yy1* (Yin Yang 1) locus. YY1 is a transcription factor that, by interacting with PRC2 (Polycomb Repressive Complex 2), inhibits the expression of muscle-specific genes such as *Myhc* or *Tnni2* (encoding TROPONIN I2). Linc-YY1 prevents the interaction of YY1 with PRC2, which supports the expression of such genes as *Myhc* or *Tnni2*, as well as miR-1, and as a result supports myogenic differentiation [77]. Opposite effect on myogenic differentiation is mediated by NEAT1 (Nuclear enriched abundant transcript 1), which is localized in the nucleus in so-called paraspeckles. NEAT1 interacts (by F3 domain, 1001-1540 bp) with EZH2 (enhancer of zeste homolog 2), a subunit of PRC2, which leads to trimethylation of H3K27 (histone H3 lysine 27) in nucleosomes associated with such genes as *Myog*, *Myhc4,* or *Tnni2* [76,81]. In addition, NEAT1 supports myoblast proliferation by downregulating the expression of *Cdkn1a* encoding p21CIP1.

Proliferation of myoblasts is further promoted by another lncRNA, i.e., Sirt1AS. It multidirectionally influences expression of gene encoding SIRT1 protein, which in turn modulates cell cycle progression. First, Sirt1AS interacts with 3′UTR of *Sirt1* gene and competes with miR-34a, which serves as a transcriptional suppressor of this gene. Next, it protects *Sirt1* mRNA from degradation by forming a duplex in the cytoplasm and in such a way promotes its translation [78]. Similarly to Neat1 action, trimethylation of muscle-specific genes is also connected with the activity of MALAT1 (Metastasis-Associated Lung Adenocarcinoma Transcript 1). This lncRNA is detected in different cell types, including cancer ones. It forms as a result of the activity of RNaseP in the nucleus. In myogenic cells, MALAT1 interacts with HP1beta and HDAC1 and recruits SUV39h1 methyltransferase to MYOD-binding sites. As result, H3K9 (histone H3 lysine K9) is trimethylated and expression of MYOD target genes, such as those encoding MYOGENIN or TROPONIN, is blocked [74]. In differentiating myogenic cells, MALAT1 is degraded by miR-181a and AGO2. In such environment, an activating complex containing SET7, a protein lysine methyltransferase, leads to monomethylation of H3K4 (histone H3 lysine 4) and promotes the expression of genes necessary for final differentiation and maturation of myogenic cells [74]. In addition, MALAT1 promotes the expression of MYOD-dependent genes by acting as a competitive endogenous sponge for miR-133 and enhancing SRF expression [73,74]. Thus, numerous lncRNAs have been already identified and described as factors engaged in myogenesis regulation. Many of them, including MALAT1, lnc-mg, H19, or linc-MD1, act as molecular sponges for miRNAs. In general, NEAT1, MALAT1, and Sirt1AS serve as examples of lncRNAs, which support myoblast proliferation and inhibit their differentiation, while Charme, CE eRNA, DRR eRNA, H19, linc-MD1, lnc-mg, linc-YY1, and Myoparr exert opposite effect on these processes.

## 4. miRNAs and lncRNAs as Tools to Improve Myogenic Differentiation

### 4.1. miRNA

The role of miRNAs in cell and tissue functioning was verified by their experimental upregulation or downregulation under various experimental settings. They were also tested as molecules that could enhance or improve myogenic differentiation of cells that could be possibly used in therapy. Among the miRNAs tested were those involved in mesoderm and/or myogenic lineage specification. Among the cells used in such tests—apart from myoblasts—were stem cells, including pluripotent stem cells (PSCs), such as embryonic stem cells (ESCs) or induced pluripotent stem cells (iPSCs). Here, we present some examples from the vast amount of data showing that by using miRNAs or lncRNAs, myogenesis could be either improved (myoblasts) or induced (PSCs).

As mentioned before, in myoblasts miR-181a targets mRNA encoding the homeobox protein HOXA11, i.e., the repressor of terminal differentiation [61]. However, this miRNA can also regulate the differentiation of other cellular lineages, such as neural or hematopoietic [82,83]. In mouse ESCs, miR-181a inhibits the translation of SIRT1, which is involved in their differentiation [84,85]. Overexpression of miR-181a in mouse ESCs led to an upregulation of the expression of *Myod1* and *Myhc2* (encoding myosin heavy chain 2), and increased proportion of mouse ESCs characterized by the presence of MYF5 [86]. Overexpression of miR-145, another differentiation-related miRNA, in C2C12 myoblasts induced *Myod1*, *Myf5*, *Myog*, and *Myhc* expression [87]. In PSCs, it impacts on factors regulating pluripotency. Transient overexpression of miR-145 in human ESCs downregulated pluripotency markers and induced the expression of genes encoding mesodermal markers, such as NODAL or MIXL1 [88]. Its overexpression in mouse ESCs also resulted in pluripotency marker downregulation and induction of *Pax3*, *Pax7*, *Myod1*, and *Myhc2* expression [86]. Overexpression of miR-27b, which is involved in mesoderm formation, led to the drop in c-MYC, NANOG, OCT4, and SMAD4, which facilitated their initial stages of differentiation [89].

As far as MyomiRs are concerned, overexpression of miR-1 and miR-133 has been shown to induce differentiation of ESCs and embryonic carcinoma cells (ECCs) toward mesodermal lineages. Cardiac and myogenic differentiation of these cells have been induced by miR-1 and inhibited by miR-133a overexpression, what was manifested by *Nkx2.5* and *Myog* expression [90,91]. However, another study in which miRNA mimics were used did not prove MyomiR overexpression as a useful tool to induce PSC myogenic differentiation. miR-1, miR-133, and miR-206 were shown to induce *Pdgfra* expression in in vitro differentiating mouse ESCs; however, myogenic factors were not upregulated [86]. This failure might be caused by the fact that MyomiRs were expressed too early, i.e., before the mesodermal specification was induced. Timely expression of miR-1 and miR-206, as it was shown for mouse satellite cells, induced myogenic differentiation by repressing *Pax7* expression [50]. Among the miRNAs other than MyomiRs, whose myogenic promoting ability was tested by their overexpression in in vitro cultured myoblasts, are miR-26a [92], miR-24 [93], miR-378 [94], and miR-19 and miR-17 [64]. The last ones were shown in vitro to be acting together to promote the expression of myogenic regulators (Table 1). When overexpressed, they induced the expression of genes encoding MRFs, MyHCs, or other myogenesis regulating genes. Some of already mentioned miRNAs were also tested in vivo, e.g., in developing embryos or regenerating muscles. Such experiment was, for example, done with miR-27b, whose overexpression in developing mouse embryos led to downregulation of *Pax3* and accelerated myogenic differentiation [60]. Thus, by manipulating the levels of miRNAs, one can improve myogenic differentiation.

### 4.2. lncRNA

Constantly growing evidence on lncRNA role in tissue function makes them another potential candidate as a tool to improve myogenic differentiation. As mentioned above, their mode of action is different, e.g., they could compete with miRNAs. The impact of many of them has been tested in overexpression experiments, both in vitro and in vivo. For example, overexpression of lnc-mg, which—as mentioned previously—inhibits miR-125b regulating IGF2, in vivo, i.e., within skeletal muscle, led to muscle hypertrophy [72]. Another lncRNA involved in miR-125b sponging is lnc-smad7 [95]. During analysis of C2C12 cell transcriptome, this RNA was identified as the one whose expression was higher in myotubes than in myoblasts [96]. Further studies documented that it promotes myoblast differentiation in vitro and improves skeletal muscle regeneration [95]. Next, overexpression of lncMD in bovine primary myoblasts significantly upregulated MyHC and MYOG both at mRNA and protein level [97]. The next one, lncMAR1, which targets miR-487b, when overexpressed in vivo, increased mouse skeletal muscle mass and force, which was associated with elevation of MYOD, MYOG, MEF2C, and MYF5 [98]. Another lncRNA—lncIRS1—sponges miR-15a, miR-15b-5p, and miR-15c-5p, which downregulate the expression of IRS1 (insulin receptor substrate 1). Thus, overexpression of lncIRS1 increased the level of IRS1 and promoted myoblast proliferation, as well as differentiation. These are only the selected examples showing that by manipulating lncRNA levels one can improve myogenic differentiation or skeletal muscle regeneration, which could be crucial for the development of future therapies.

## 5. Physical Activity Impact on miRNA and lncRNA

Thus far, the exact molecular mechanisms responsible for body adaptation to exercise have not been defined. However, it seems that physical activity can regulate the expression of various genes by affecting DNA methylation [99], histone modifications [100], miRNA, or lncRNA expression [101,102,103,104,105]. Analyses of material obtained from human skeletal muscle biopsies or body fluids have shown that various training protocols and body adaptations differently impact miRNA and lncRNA levels. Here, we will describe miRNA and lncRNA changes during resistance and endurance training.

Resistance training refers to static strength exercises that require the use of resistance, e.g., lift- or body weight exercises, to induce muscle contraction. Excessive overloads cause muscle fiber microdamages, which can activate the satellite cells, stimulating their proliferation and differentiation. The effect of resistance training is to increase muscle mass (hypertrophy) and strength. In contrast to resistance training, endurance training includes dynamic exercises, often called ‘cardio’ or aerobic, such as running, swimming, or cycling. Endurance training causes a wide range of changes, mainly in the cardiovascular and respiratory systems, rarely leading to extensive microdamages in muscle fibers. Short-term, high-intensity training is usually called acute, and long-term, moderate-intensity workouts are called chronic. High-intensity interval training (HIIT) is a short-term (30 min) workout involving periods of high-intensity aerobic exercise combined with moderate-intensity exercise. In addition, HIIT can include any type of exercise, including cycling, running, or strength exercises. Moderate-intensity continuous training (MICT) consists of 30–60 min of aerobic exercise that is less intensive than in HIIT.

### 5.1. Expression of miRNAs in Muscle Tissue after Resistance or Endurance Training

The first data focused on changes in miR-1, miR-133a, and miR-206 expression in mouse soleus and plantaris muscles analyzed after resistance training showed that before overload, the level of miR-1 and miR-133a was the same in both muscles. miR-206 expression, however, was sevenfold higher in the soleus, as compared to plantaris, muscle [106]. This was also the first evidence that different muscles may vary in the expression of individual miRNAs. After overload, expression of miR-1 and miR-133a dramatically decreased, whereas miR-206 was unchanged. Similar downregulation of miR-1 was reported in muscle biopsies from young men after resistance training (Table 3) [101]. Downregulation of miR-1 was also confirmed in resistance-trained men and women [107]. The decrease in miR-1 level was accompanied by an increase in *IGF-1* expression [107]. This increase was associated with activation of the mTORC1 pathway. Activation of the mTORC1 pathway increases the synthesis of muscle proteins such as MYOGENIN and MyHCs. As a result, an increase in muscle mass occurs. Furthermore, downregulation of miR-133a, miR-133b, miR-206, miR-208a-5p, and miR-499 was shown to be caused by acute resistance exercise or strength-endurance training (HIIT or MICT with resistance training) (Table 3) [102,103,108]. What is more, animal studies have shown that downregulation of miR-1 and miR-206 substantially enhances satellite cell proliferation [50]. All of these data are consistent with the results showing that human resistance training leads to increased muscle protein synthesis and intensive satellite cell proliferation, 1–4 days after exercise [109,110,111]. Next, downregulation of miR-1, miR-133, miR-206, and miR-499 is associated with muscle hypertrophy [108,112]. However, decrease in miR-1, miR-133a, miR-133b, miR-206, and miR-486-5p was not observed in biopsies of vastus lateralis muscles of young and old men after a short (2 h) acute resistant training session (Table 3) [113]. Such discrepancy may result from the different training program or premature collection of muscle biopsies.

In the case of endurance exercise, miRNA level in muscle depends on both the duration (short-term vs. long-term) and intensity (high vs. moderate) of training. Transcription level of miR-1, miR-133a, miR-133b, and miR-181a was increased after short-term (60 min), high-intensity cycle ergometer activity in untrained males (Table 3) [115,116]. Furthermore, the increase in miR-1 and miR-133a expression, observed immediately after intense endurance exercise, was proportional to the increase in MYOD, MYOGENIN, and MRF4 synthesis. Such MRF changes are likely to result from the increase in MyomiR expression [44,124]. Contrasting data come from experiments that examined the results of long endurance training at moderate intensity [114]. Decreased expression of miR-1, miR-133a, and other miRNAs (miR-28, miR-144, and miR-455), in leg muscles, was reported after prolonged (6 weeks) moderate-intensity cycling in young men [114]. Moreover, another analysis of muscles of young males showed a decrease in the miR-1, miR-133a, miR-133b, and miR-206 after long (12-week) endurance training on a cycle ergometer (Table 3) [115].

### 5.2. Expression of c-miRNA in the Bloodstream after Resistance or Endurance Training

miRNA can be also detected in body fluids. Such molecules are described as circulating miRNAs—c-miRNAs. c-miRNAs were detectable in 12 human body fluids, i.e., serum, plasma, saliva, tears, urine, amniotic fluid, colostrum, breast milk, bronchial lavage, cerebrospinal fluid, peritoneal fluid, pleural fluid, and seminal fluid, isolated from healthy individuals [125,126,127,128,129]. Depending on which organ is more sensitive to the training protocol, miRNAs circulating in the bloodstream may come, for example, from damaged skeletal or cardiac muscle, vascular endothelium, lungs, or liver. Presence of c-miRNAs may play an important role in stimulating a faster exercise-induced immune response, leading to proper muscle regeneration. c-miRNAs can bind to Toll-like receptors and induce cytokine secretion by immune cells [130]. Other data documented intercellular transfer of miRNAs by exosomes between immune cells, leading to gene expression changes in recipient cells [131]. After 3 days of resistance training, increased expression of miR-149 and decreased miR-146a and miR-221 were found in males’ serum (Table 3). In contrast, there were no changes in the levels of muscle-specific c-miRNAs such as miR-1 and miR-133 [118]. This effect was found to be associated with improved male adaptation to training load and therefore with a lower proportion of microinjuries from which MyomiRs (miR-1 and miR-133) could be released. On the other hand, it is possible that MyomiRs are not secreted into the blood by passive transport but by a more complex, selective mechanism. However, this issue requires more detailed studies.

Marathon training is a type of endurance exercise that can cause extensive muscle damage [132]. After half-marathon, plasma levels of miR-1, miR-133a, and miR-206 were significantly upregulated (Table 3) [119]. Additionally, a significant increase in the expression of miR-1, miR-133, and miR-30a was observed in the plasma of professional marathon runners [120]. Moreover, the level of c-miRNA associated with inflammatory processes differed significantly after short- (10 km) and long-distance (marathon) runs (Table 3) [121]. Those changes in MyomiRNA levels after marathon can be caused by intensive skeletal and/or heart muscle response and by following regeneration. It should be taken into consideration that changes in c-miRNA profile depend on the training protocols, preparation to training overloads, time of the workout, or range of muscle injuries. Specific training protocols are known to affect different signaling pathways connected with angiogenesis, synaptogenesis, inflammation, muscle recovery, etc. As mentioned before, several miRNAs are expressed both in skeletal and cardiac muscle, e.g., miR-1, miR-133a, miR-208b, miR-486, and miR-499a/b [47,54,133]. It is possible that after intensive exercise, miRNAs detected in blood may originate not only from skeletal but also cardiac muscle. Simultaneous analysis of miRNAs, which are specific exclusively to skeletal (miR-206, miR-133b) and cardiac (miR-208a) muscles, may be the solution to this problem. Further large-scale studies should be performed to answer the question of whether c-miRNAs could be markers suitable for monitoring skeletal muscle function or recovery.

### 5.3. Expression of lncRNAs in Muscle Tissue after Resistance or Endurance Training

Until today, only a few publications have presented analysis of the dynamics of lncRNA expression after physical exercise in humans. It was shown that in young men and women, lncRNA expression is exercise-specific and related to the training program, i.e., HIIT (high-intensity interval training), endurance training, resistance training, and combined-exercise training [104] (Table 4). HIIT and endurance training resulted in H19 upregulation, which was also observed, together with downregulation of let-7 during human myoblast differentiation in vitro [134,135]. Moreover, H19 is also known to increase insulin sensitivity by activating AMPK in skeletal muscle. Activation of AMPK promotes glucose uptake and fatty acid oxidation and enhances glycogen storage capacity in skeletal muscle [136]. Another previously described lncRNA, i.e., linc-MD1, which promotes differentiation of mouse and human myoblasts by sponging miR-133/miR-135 [36], was also upregulated after HIIT session in men and women muscles. Animal studies documented that MALAT1, H19, lnc-mg, linc-MD1, linc-YY1, and Sirt1-AS were overexpressed in muscles damaged due to physical exercise [137]. Moreover, positive correlation between aforementioned lncRNAs and skeletal muscle regulatory factors (MYF5, MRF4, MYOGENIN, HIF-1α, ANGIOPOIETIN 1) was observed [137]. Additionally, lncRNA AC010198.2 upregulation after HIIT was described [104]. This lncRNA is miR-143 negative regulator. Downregulation of miR-143 in bovine muscle satellite cells increased muscle differentiation [138] and in porcine muscles reduced slow muscle fiber formation [139]. To sum up, these data have demonstrated that HIIT and endurance training improve glucose metabolism and may influence fiber type formation by lncRNA controlled mechanism. Next, five other lncRNAs were most significantly upregulated (AC009754.2 AL009178.2, AC009041.2, AC012636.1) and five were most significantly downregulated (AC063919.1, AC073389.2, AC116535.1, AC018467.1, and AL137127.1), after resistance training [104]. Changes in this lncRNA expression were related to extracellular matrix organization, sprouting angiogenesis, positive regulation of PI3K (a pathway inducing skeletal muscle hypertrophy), positive regulation of intracellular signal transduction, striated muscle contraction, and myofiber innervation [104].

Analysis of 242 differently expressed lncRNAs in trained vs. sedentary elderly men allowed one to pinpoint transcriptional networks affecting processes connected with the beneficial effect of exercise training [105]. Thus, lncRNA GAS5 was significantly downregulated in trained men and was linked to anti-inflammatory effect and increase in muscle mass and strength. Additionally, cancer studies indicated that GAS5 knockdown upregulates proangiogenic factor—VEGFA [140]. Post-exercise downregulation of GAS5 probably induces new capillary formation in muscle. Next, downregulation of SNHG12 coupled with an upregulation of its target miR-181a was observed in trained elderly. This may result in higher muscle mass and mitochondrial dynamics in skeletal muscle [141,142]. Additionally, higher expression of MALAT1 in muscles after regular exercise was described [105]. Apart from its role in myogenic differentiation, i.e., modulation of MYOD transcriptional activity, it was shown that MALAT1 influences synapse formation by regulating the expression of genes involved in synaptogenesis, e.g., *NLG1* encoding NEUROLIGIN1 and *CADM1* [143].

Taken together, lncRNAs are important players in various biological processes related to physical activity adaptations. Their expression seems to be exercise-specific, but this field is continuously developing. Further studies are needed to verify whether the levels of lncRNAs depend on: sex, age, body weight, BMI, physical capacity, time, or training intensity.

## 6. Conclusions

We hope that the examples of miRNAs and lncRNAs described in this article prove that these molecules could serve as a powerful tool to enhance myogenic differentiation. They are also effective regulators of postexercise adaptation and recovery, and in the future they might be used to assess muscle damage or wasting caused by intensive training or disease. However, further studies are needed to fully understand their role and make them applicable in diagnostics or treatment.

## Figures and Tables

**Table 1:** Selected miRNAs involved in myogenic differentiation.

miRNA	Selected Target Factors	Function	Selected References
miR-1	PAX3, PAX7, HDAC4, and ID1-3	promotes myoblast differentiation	[50,51,52,53,54]
miR-206	PAX3, PAX7, NOTCH3, IGFBP5, HDAC4, and ID1-3 DNA POLA1	promotes myoblast differentiation	[49,50,51,52,53,54,55,57,59]
miR-133a/b	cyclin D1, SRF	supports myoblast proliferation, inhibits differentiation	[47,59]
miR-27b	PAX3	promotes myoblast differentiation	[60]
miR-486	PAX7	promotes myoblast differentiation	[51]
miR-181a	HOXA11	required for terminal stages of myogenic differentiation	[61]
miR-214	N-RAS	promotes myoblast differentiation, limits myoblast proliferation	[59,62,63]
miR-17/19	MRFs	regulates myoblast differentiation	[64]

**Table 2:** Selected lncRNAs involved in myogenic differentiation.

lncRNA	Selected Target Factors	Function	Selected References
CE eRNA	MYOD	promotes myoblast differentiation	[69] [70]
DRR eRNA	MYOG	promotes myoblast differentiation	[71]
H19	let-7	promotes myoblast differentiation, inhibits myoblast proliferation	[35,65,66]
linc-MD1	miR-133, miR-135,	promotes myoblast differentiation,	[36]
lnc-mg	miR-125b, miR-351-5p	promotes myoblast differentiation, inhibits myoblast proliferation	[72]
MALAT1	miR-133, with HP1beta, HDAC1, and SUV39h1, blocks MYOD target genes, such as MYOG and TNNI2	promotes myoblast differentiation but also suppresses it, depending on interactions	[73,74]
Myoparr	MYOG, miR-133b, miR-206, and H19	promotes cell cycle withdrawal and myoblast differentiation	[75]
NEAT1	MYOG, MyHC4, TNNI2, and p21CIP1	promotes myoblast proliferation, inhibits differentiation	[76]
linc-YY1	YY1	promotes myoblast differentiation	[77]
Sirt1-AS	SIRT1, miR-34	supports myoblast proliferation	[78]

**Table 3:** miRNAs associated with different types of training. Listed in order of appearance within the text. Bold font emphasizes striated muscle-specific miRNAs.

TRAINING TYPE	Upregulated miRNAs	Downregulated miRNAs	Reference
**miRNAs detected in skeletal muscles (biopsies)**			
Acute resistance training (3/6 h)	-	**miR-1**	[101]
Chronic resistance training (12 weeks)	-	**miR-1**	[107]
Acute resistance training (6 h)	miR-423-5p	miR-16-5p, miR-23b-3p, miR-24-3p, miR-26a-5p, miR-26b-5p, miR-27a-3p, miR-27b-3p, miR-29a-3p, miR-29c-3p, miR-30a-5p, miR-30d-5p, **miR-133a, miR-133b**, miR-95-3p, miR-107a, miR-126-3p, miR-140-3p, miR-181a-5p, miR-324-3p, and miR-378a-5p	[102]
HIIT or MICT + resistance training (1/3 h)	-	**miR-133a**, miR-378, and **miR-486**	[103]
Endurance with overload training (90 min)	-	**miR-1-3p, miR-206,** **miR-208a-5p, and miR-499**	[108]
Long-term endurance training (6 weeks)	-	**miR-1, miR-133a**, miR-101, miR-455, miR-144, miR-15b, miR-26b, miR-28, miR-29b, miR-338, miR-92, miR-98, miR-451, and miR-589	[114]
Long-term endurance training (moderate-intensity, 12 weeks)	-	**miR-1, miR-133a, miR-133b, and miR-206**	[115]
Short-term endurance training (high-intensity, 60 min)	**miR-1, miR-133a, miR-133b, and miR-206**	-	[115]
Short-term endurance training (high-intensity, 60 min)	**miR-1, miR-133a/b,** and miR-181a	miR-9, miR-23a, miR23b, and miR-31	[116]
**circulating miRNAs (c-miRNA) detected in the bloodstream (plasma or serum)**			
Long-term endurance training (1-3h/session, 90 days)	miR-20a, miR-21, miR-146a, miR-221, miR-222, miR-21, and miR-146a	-	[117]
Acute resistance training (1 h/1 day/3 days)	miR-149	miR-146a, miR-221	[118]
Half-marathon	**miR-1, miR-133a,** **miR-206**	-	[119]
Endurance training (10 weeks) + marathon	**miR-1, miR-133a**, and miR-30a	miR-26a, miR-29b	[120]
10 km run + half-marathon + marathon (1-month intervals)	let-7d-3p, let-7f-2-3p, miR-125b-5p, miR-132-3p, miR-143-3p, miR-148a-3p, miR-150-5p, miR-223-3p, miR-223-5p, miR-29a-3p, miR-34a-5p, miR-424-3p, and miR-424-5p	-	[121]
Long-term endurance training (20 weeks)	miR-142-3p, miR-221-3p, miR-126-3p, miR-146a-5p, and miR-27b-3p	**miR-486-5p**, let-7b-5p, miR-29c-3p, let-7e-5p, miR-93-5p, miR-7-5p, miR-25-3p, miR-92a-3p, and miR-29b-3p	[122]
Cardiopulmonary exercise (3 min) and acute endurance training (60 min)	miR-21	miR-20a	[123]

**Table 4:** lncRNAs associated with different type of training.

Training Type	Upregulated miRNAs	Downregulated miRNAs	Reference
**lncRNAs detected in skeletal muscles (biopsies)**			
HIIT (12 weeks)	H19, linc-MD1, AC010198.2, AP000688.2, CLLU1, FEZF1-AS1, and AC007222.1	AC131934.1, AC099754.1, AC098936.1, AC010105.1, and AC018467.1	[104]
Resistance training (12 weeks)	AC009754.2, AL009178.2, AC009041.2, AC132872.4, and AC012636.1	AC063919.1, AC073389.2, AC116535.1, AC018467.1, and AL137127.1	[104]
Combined resistance and endurance training (12 weeks)	AL513318.2, AL359704.3, AL136366.1, AC015878.1, and USP2-AS1	LINC-PINT, AL662844.4, AC022706.1, LINC01091, and AC012378.2	[104]
Regular endurance or resistance training (at least 30 years)	MALAT1, SNHG14, SNHG16, and TP73-AS1	GAS5, SNHG12, HOTAIRM1, ZFAS1, RP11-48O20.4 (LINC01133), HOXD-AS1 (HAGLR), SNHG12, EMX2OS, SNHG15, LINC00152 (CYTOR), SNHG1, and HOXC-AS1	[105]

## Data Availability

Not applicable.
